# The perceptual continuity field is retinotopic

**DOI:** 10.1038/s41598-019-55134-6

**Published:** 2019-12-11

**Authors:** Thérèse Collins

**Affiliations:** Integrative Neuroscience and Cognition Center, Université de Paris & CNRS, 45 rue des Saints-Pères, 75006 Paris, France

**Keywords:** Perception, Psychology

## Abstract

Visual perception is systematically biased towards input from the recent past: perceived orientation, numerosity, and face identity are pulled towards previously seen stimuli. To better understand the brain level at which serial dependence occurs, the present study examined its spatial tuning. In three experiments, serial dependence occurred between stimuli occupying the same retinal position. Serial dependence between stimuli at distant retinal locations was smaller, even when the stimuli occupied the same location in external space. The spatial window over which serial dependence occurs is thus retinotopic, but wide, suggesting that serial dependence occurs at late stages of visual processing.

## Introduction

Our impression of the visual world starts with the patterns of light that fall on our retinae and launch a cascade of events that result in the subjective experience of a stable, continuous world. How this stability arises is a crucial question in visual neuroscience, given that the input is highly variable: changes of lighting due to modifications in the world (such as the sun going behind a cloud), changes in aspect and size due to body movements (such as moving towards or away from an object), spurious changes in object position due to eye movements.

The neural and psychological mechanisms that underlie the transformation of variable visual input to stable visual experience remain to be fully uncovered. One way that perceptual stability may be achieved is by the integration of sensory evidence over time, such that the immediate content of perception is the result not only of current visual input, but also takes recent stimulus history into account. Such integration is quantified by a phenomenon called serial dependence^1^. Fischer & Whitney^2^ asked participants to report the orientation of a tilted Gabor patch, and observed that responses were pulled in the direction of previously-seen patches. Serial dependence has been found to occur for a wide range of visual attributes such as numerosity^3,4^, motion^5^ and position^6^. Perception of higher-level stimuli are also subject to serial dependence. For example, face identity^7^, attractiveness^8^, and gaze orientation^9^; and even summary statistics of a visual scene^10^ – although in the former examples, serial dependence may be driven by lower-level features of the visual stimuli composing the faces or ensembles.

Determining the tuning characteristics of serial dependence is crucial to fully understanding the phenomenon and its neural substrates. Serial dependence is temporally tuned (reaching back in time by a few tens of seconds), feature-tuned (for example, only nearby orientations exert an influence on subsequent orientation perception), and spatially tuned. Fischer & Whitney^2^ measured to what extent serial dependence depended on the physical distance between successive stimuli, and found that even with relatively large distances, there was still an effect of previous stimuli on current perception. They termed the spatial window within which current perception is influenced by past stimuli the continuity field (CF). The CF is roughly circular, with a radius of ~15 degrees of visual angle (dva). They also tested whether the field was defined in retinotopic or spatiotopic coordinates by asking participants to change fixation locations between trials. Thus, on some trials, successive stimuli occupied the same position on the retina but not in space, while on others, successive stimuli appeared at the same location on the screen, but at different retinal locations due to intervening eye movements. They found a significant serial dependence in both of these conditions, and concluded that there was therefore a spatiotopic component to the continuity field. However, the distance between tested positions (13 dva) was smaller than the continuity field. Thus, serial dependence in trials labelled “spatiotopic” could, in fact, be due to a large retinotopic continuity field.

The goal of the current set of experiments was to test whether the continuity field is spatiotopic or retinotopic. We asked participants to match the orientation of a Gabor patch and examined serial dependence between trials.

Because the coordinate frame and receptive field size have been characterized for numerous visual brain areas, an accurate description of the coordinate frame and spatial extent of the continuity field may be a pointer towards candidate brain areas sub-serving visual stability.

## Results

**Figure 1:**
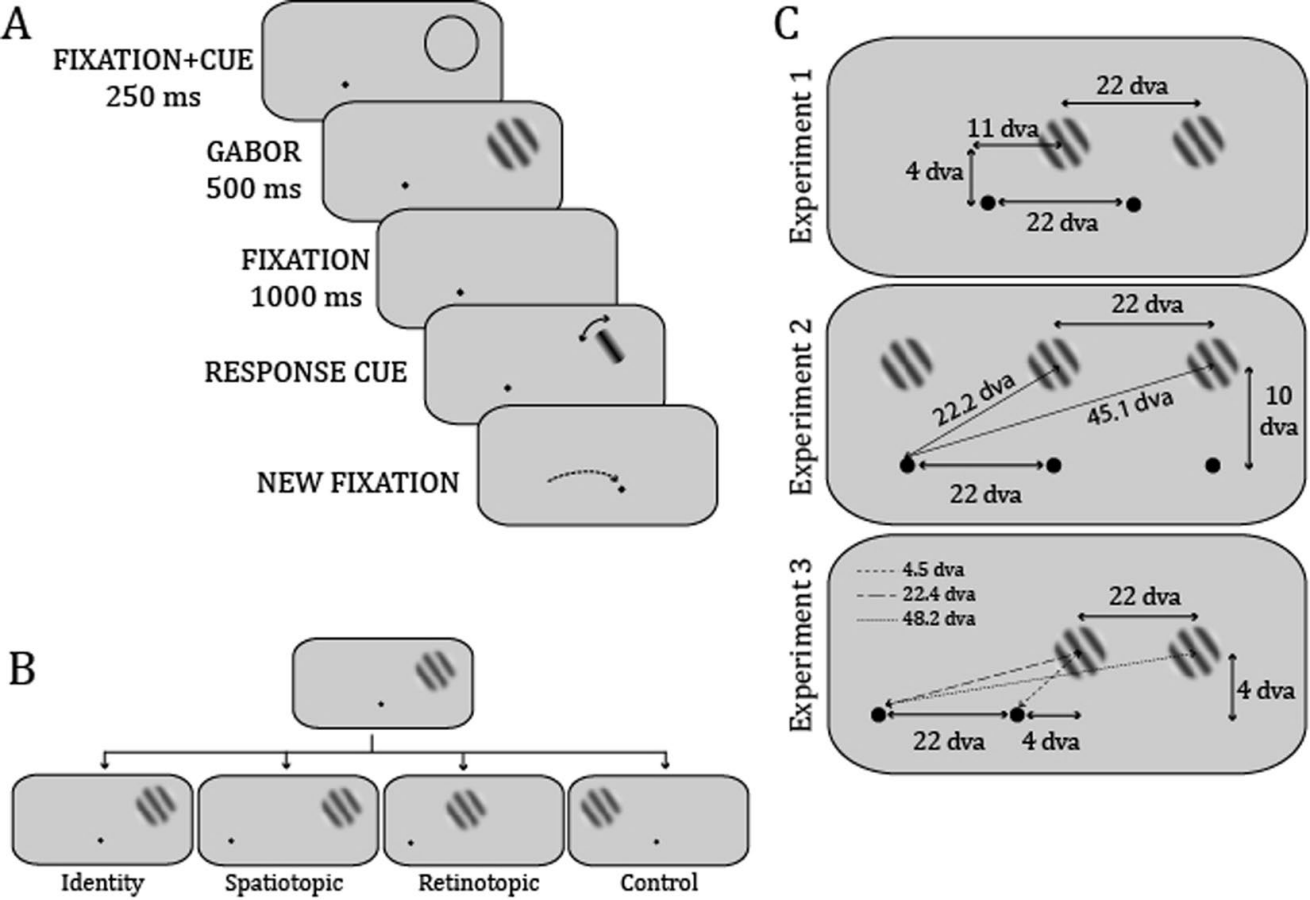
(**A**) Procedure. A fixation and, in experiments 2–3, a cue, appeared for 250 ms, followed by a Gabor patch for 500 ms, a 1000-ms blank, and the response cue. Subjects adjusted the response cue until it matched the Gabor patch, then pressed on the space bar to validate their response and go to the next trial. (**B**) Conditions given a previous trial with a central fixation point and a Gabor patch on the top right. Identity: same fixation point and Gabor patch position on the screen between previous and current trials, i.e. no saccade between trials; Spatiotopic: new fixation point but same Gabor patch position on the screen; Retinotopic: new fixation and new Gabor patch position on screen, but relative positions of fixation point and Gabor patch identical between previous and current trials; Control: new Gabor patch position on screen, fixation point and Gabor patch at different relative positions between trials. (**C**) Spatial configuration of Experiments 1, 2 and 3 (top, middle, bottom panels respectively).

Participants viewed a single Gabor patch on each trial and reported its orientation by adjusting a bar (Fig. 1A). Between trials, patches could occupy the same location on the screen (spatiotopic condition), the same location on the retina (retinotopic), both (identity), or neither (control) (Fig. 1B). Serial dependence was quantified by examining the relationship between the relative orientation between two successive trials (i.e. the circular distance between current and previous Gabor patch orientations) and response error (i.e. the circular distance between Gabor patch orientation and response). A first derivative of Gaussian (DoG) was fitted to each condition (see Methods); the amplitude of the DoG quantified serial dependence.

**Figure 2:**
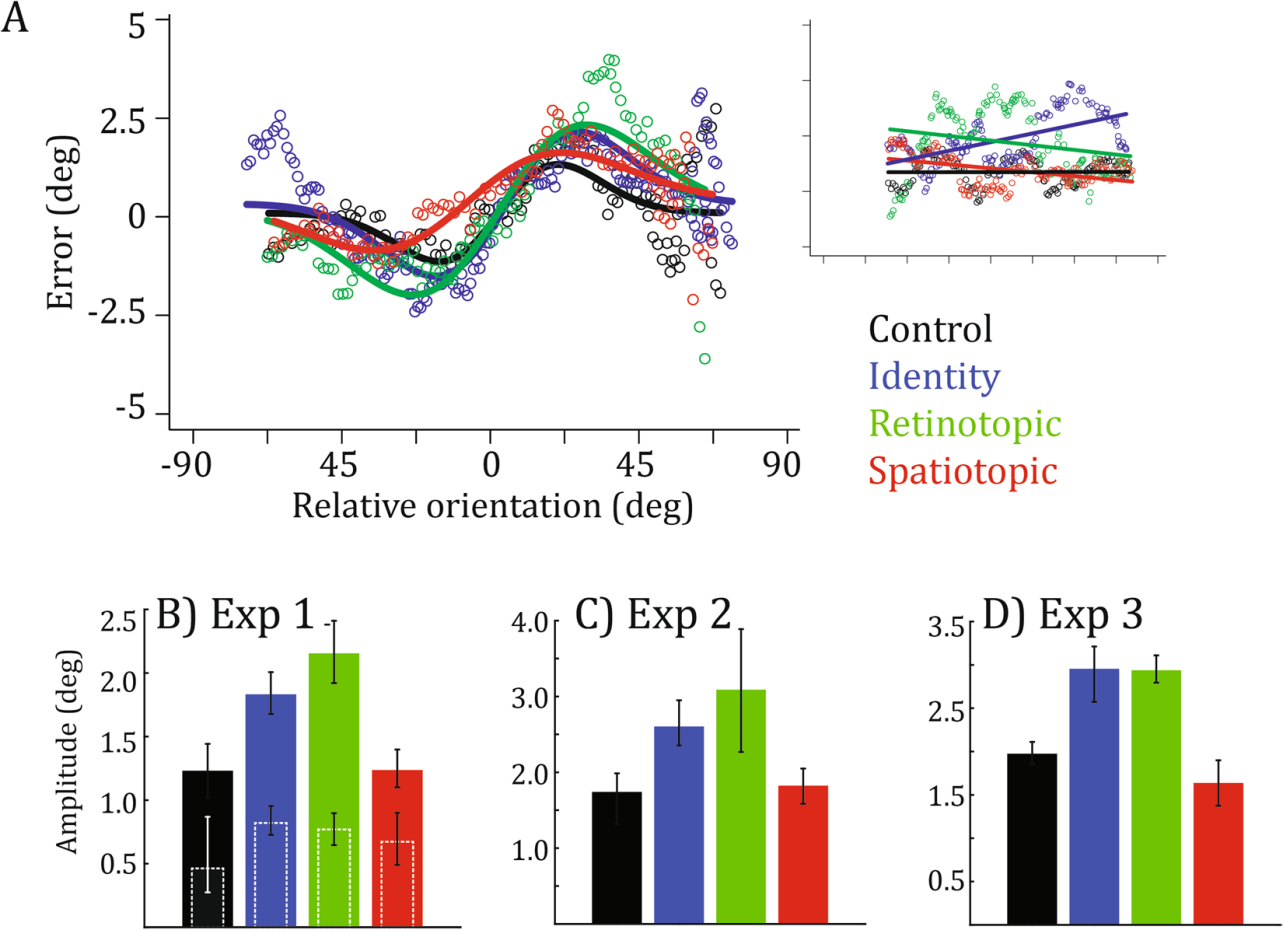
(**A**) Error as a function of relative orientation between previous and current trials, for each of the four conditions, in Experiment 1. The inset shows error as a function of relative orientation between the current trial and one trial in the future. (**B**–**D**) Amplitude (mean ± 95% confidence intervals) of the DoG for each of the four conditions, in Experiments 1–3. Dashed outlines in Experiment 1 show amplitude of serial dependence between 2-back trials.

Figure 2A presents error as a function of relative orientation and the DoG fits for each condition in the first experiment, on data pooled over 12 participants. Figure 2B represents the amplitude of the fitted DoGs for each condition. Error bars represent 95% confidence intervals estimated by a permutation test, thus, non-overlapping error bars represent significant differences between conditions. Amplitude was similar between control (1.23, 95% CI = [1.00–1.43]) and spatiotopic conditions (1.26, [1.11–1.39]), and higher for both identity (1.84, [1.68–2.01]) and retinotopic conditions (2.16, [1.91–2.40]).

There was a tendency for two-back trials to influence perceived orientation, but it was smaller than one-back trials and did not differ between trial types (dashed outlines in Fig. 2B; 95% confidence intervals in the control condition: [0.30–0.83]; identity: [0.71–0.94]; retinotopic: [0.66–0.90]; spatiotopic: [0.50–0.87]). The inset of Fig. 2A shows error as a function of relative orientation between the current trial and one trial in the future. The relationship was not captured by DoGs (linear fits are shown for illustrative purposes), and serves as a check on spurious inter-trial correlations.

Results were strikingly similar in a second group of participants (n = 11) with a slightly modified paradigm (see Fig. 1C and Methods): amplitude was comparable between control (1.74, [1.32–1.99]) and spatiotopic conditions (1.82, [1.59–2.05]), and higher for both identity (2.60, [2.36–2.95]) and retinotopic conditions (3.09, [2.27–3.89]) (Fig. 2C).

**Figure 3:**
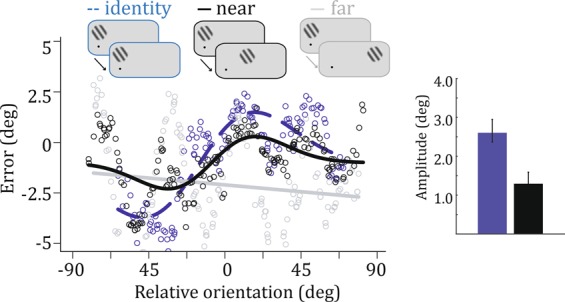
Error as a function of relative orientation depending on distance between previous and current Gabor patches. Identity condition: zero dva apart; near condition: 22 dva apart; far condition: 44 dva apart. The grey line illustrates a linear fit to the far data (that could not be fit by a DoG). Inset: Amplitude (mean ± 95% confidence intervals) of the DoG for identity and near conditions.

The distance between successive Gabor patches influenced the strength of serial dependence. Quantification of the distance effect was made possible by the spatial set-up of Gabor patches in Experiment 2 (Fig. 1C), by comparing trials in which the distance between successive patches was 0 (identity), 22 or 44 dva. Consider a trial in which both the fixation and the Gabor were presented at the leftmost locations, followed by a trial with the same fixation location but a Gabor at the middle location. The distance between the center of the putative retinotopic continuity field (assumed to be centered on the previous Gabor path location) and the new Gabor is 22 dva; thus, this Gabor probably falls near its edge, and, if the CF is indeed retinotopic, one would expect smaller serial dependence. Consider a trial in which both the fixation and the Gabor were at the leftmost locations, followed by a trial with the same fixation location but a Gabor at the far right location. The distance between the putative retinotopic CF and the new Gabor is 44 dva. In the near condition (22 dva distance between successive patches), serial dependence was similar to the control condition (of which the near trials are a subset): 1.29 [1.05–1.59], and smaller than the identity condition (2.60, [2.36–2.95]). In the far condition (44 dva), data did not follow a DoG shape and could not be fit with the model (Fig. 3). Although there is quite some variability in the far condition, there does not seem to be a simple relationship between perceived orientation and relative orientation between trials, suggesting that only nearby Gabor patches elicit serial dependence. (For illustrative purposes, Fig. 3 shows a linear fit to the far data).

Finally, the greater serial dependence in the retinotopic condition relative to the spatiotopic condition observed in the previous experiments was not due to changing hemifields. Indeed, in the spatiotopic condition in both first and second experiments, the two successive stimuli were presented in opposite hemifields, whereas in the retinotopic condition, they were in the same hemifield. The smaller serial dependence in the spatiotopic condition may be due to the fact that orientation information was degraded when passing through commissural fibers. In a third experiment (n = 5), Gabor patches were always presented in the right hemifield (Fig. 1C). Results (Fig. 2D) showed again that amplitude was similar between control (1.97, [1.85–2.11]) and spatiotopic conditions (1.64, [1.37–1.90]), and higher for both identity (2.95, [2.57–3.21]) and retinotopic conditions (2.94, [2.79–3.11]).

## Discussion

In three experiments, perceived orientation was pulled towards previously seen stimuli. This serial dependence was maximal when current and previous stimuli shared the same location on the retina, and decreased when the retinal distance between successive stimuli increased. Although the continuity field is best characterized as retinotopic, its size is relatively large: serial dependence was observed even when successive stimuli were 22 dva apart (but not when they were 44 dva apart).

What are the mechanisms by which previous stimuli could exert an influence on current perception?

One possibility is that orientation-selective cells in visual areas could be re-activated by memory representations of previous stimuli. However, the decay time, spatial extent and topographic organization of such templates is unknown, making it difficult to derive quantitative predictions from the memory hypothesis. Furthermore, the retinotopy of the CF makes this memory-template reactivation hypothesis unlikely, because such an effect would be independent of space (unless the template were spatially specific and retinotopic, in other words, visual). The fact that there is a field, and even more so its retinotopy, argues in favor of a visual/perceptual origin of serial dependence.

The issue of whether serial dependencies occur at perceptual or post-perceptual/decisional stages was recently examined by Fritsche, Mostert & de Lange^11^ and Cicchini, Mikellidou & Burr^12^. By inducing serial dependence on one stimulus, and then asking subjects to compare that stimulus with another that had not been preceded by an inducing stimulus, Fritsche *et al*. claimed that serial dependencies in perception were repulsive, and that attraction occurred at the decisional stage. However, Cicchini *et al*.^12^ extended their methods to cover a wider range of inducer-stimulus differences and showed that when orientations were similar, perceptual serial dependencies were attractive, and that a small repulsive effect occurred only for larger differences (significantly for Fritsche *et al*. but not significantly, although with a similar effect size, in Cicchini *et al*.). Furthermore, several studies have shown that serial dependence does not depend on subjects emitting a response: stimuli that subjects do not respond to still exert an effect on the perception of subsequent stimuli^2,4^. These results also argue in favor of a visual/perceptual level for serial dependence.

If serial dependence is a visual/perceptual effect, a potential mechanism could be lingering activity in orientation-selective neurons in striate or extrastriate cortex. This seems unlikely as the decay time of orientation-specific cell activity (i.e. return to baseline after stimulus offset) is much faster (<100 ms; e.g.^13^) than the inter-stimulus-interval in serial dependence studies (on the order of several seconds).

Barring the timing issue, intra-area lateral connections between neurons with similar orientation tuning could cause serial dependence. This hypothesis can make quantitative predictions as to the CF because the spatial extent of such lateral connections has been estimated, at least in V1 in which they extend the classic receptive field of orientation-selective cells by up to 3 dva^14^. This is much smaller than the extent of the continuity field, therefore, lateral connections alone cannot explain long-range spatial interactions such as those seen here. Although the topological organization and spatial extent of lateral connections in extrastriate areas has not be quantified, it seems unlikely that they could explain the very large distances across which serial dependence operates.

Feedback connections from extrastriate visual areas onto orientation-selective cells in V1 are topographic and extend classic receptive fields to regions of space of up to ~15 dva in diameter^14^, which is smaller than the extent of the continuity field measured by Fischer & Whitney^2^ and in the present experiment (in which serial dependence was found between stimuli 22 dva apart), again making it unlikely that these connections mediate serial dependence for orientation. Furthermore, the largest receptive fields in extrastriate cortex are ~6 dva in diameter^15,16^, which again makes them an unlikely candidate for the long-range spatial interactions seen here.

The size of the CF is more similar to inferior temporal cortex receptive fields, which can extend up to 50 dva^17^. One speculative schema for serial dependence could be that orientation-selective neurons in striate and extra-striate cortex activate inferior temporal neurons with a wide receptive field. The activity from these temporal neurons would then feed back to lower levels, conferring elevated activation to neurons with similar orientation tuning in lower visual areas across a wide spatial range. When subsequent oriented stimuli are presented, they activate neurons selective for the (real) stimulus orientation, but perception is a population response. The population will be pulled towards recently seen orientations, even more so when these are close to the current orientation, because orientation is topographically represented.

In sum, the present results show that the spatial tuning of serial dependence is broad and retinotopic.

## Methods

### Subjects

Healthy human adults were contacted via the subject pool maintained by the Relais d’Informations sur les Sciences Cognitives (UMS ENS-CNRS 3332), and participated in the experiments in exchange for payment (10€/hour). All had normal, uncorrected vision, and reported no neurological or visual deficits. All gave written informed consent. The experiments were carried out in accordance with the Declaration of Helsinki and received ethical approval from the local ethics board (Conseil d’Evaluation Ethique pour les Recherches en Santé, Paris Descartes University). The number of subjects was 12 (7 men, aged 18–42 years) in experiment 1, 11 (3 men, aged 21–39 years) in experiment 2, and 5 (2 men, aged 24–39 years) in experiment 3.

### Stimuli

Fixation dots (diameter of 0.25 dva) could appear at one of several locations on the screen (Fig. 1). Gabor patches were 100% contrast, 2 cpd of spatial frequency. On any one trial, only one fixation and one Gabor patch were presented. The spatial configuration differed between experiments and is illustrated in Fig. 2.

In experiment 1, when subjects were fixating on the left dot, the Gabor patch always appeared 11 dva to the right and 4 dva above. When subjects were fixating on the right dot, the Gabor patch could appear 11 dva to the left or right, and 4 dva above. Thus, Gabors were all equidistant from fixation and of the same size (2 dva in diameter). In experiments 2 and 3, all combinations between fixation positions and Gabor patch positions were possible, thus, Gabors could more more or less eccentric. The closest Gabor was 2 dva in diameter; for other eccentricities size was m-scaled to ensure roughly similar cortical representation^18^.

### Procedure

Subjects were asked to fixate a red dot on the screen. When correct fixation was detected for more than 200 ms, the dot turned black and the trial began. The dot stayed on the screen for 250 ms. In Experiments 2 and 3, a cue indicated the location of the upcoming Gabor, because serial dependence has been shown to be greater at attended locations^2^. After 250 ms of fixation, the Gabor patch was presented for 500 ms, followed by 1000 ms of only the fixation dot, and finally the response cue. Subjects were required to maintain fixation throughout, but eye position was checked up until the Gabor patch offset only. If eye position differed from the fixation dot by more than 1.5 dva, the trial was cancelled and later rerun. The orientation of the Gabor patch and the response cue were independently selected randomly from the interval −90° to 90°. Subjects could rotate the response cue clockwise or counterclockwise by using the left and right arrows on the keyboard (each button press rotated the cue by 1 deg), and when they were satisfied that the cue matched the Gabor patch, they validated their response by pressing on the space bar. After an interval of 500 ms, the next trial fixation dot appeared.

The number of trials and sessions was as follows: in experiment 1, each subject ran a single 540-trial experimental session that took approximately 45–60 minutes to complete. In experiment 2, each participant ran 1800 trials in 6 sessions that could be separated by up to a few days. Each session was 300 trials long and took approximately 30–45 minutes to complete. In experiment 3, each participant ran 800 trials in 2 sessions that could be separated by up to a few days. Each session was 400 trials long and took approximately 45 minutes to complete.

### Eye movement recording and apparatus

Viewing was binocular. Movements of the right eye were monitored with an Eyelink 1k (SR Research, Mississauga, Ontario, Canada) at 1000 Hz sampling rate. At the beginning of a session, the Eyelink was calibrated with the standard 9-point Eyelink procedure. Before each trial, fixation was checked and if the measured value was greater than 1.5 dva, a new calibration was initiated. Calibration was also automatically renewed every 100 trials. For offline analyses, eye movement samples were smoothed with SR Research’s proprietary algorithms. Instantaneous velocity and acceleration were computed for each data sample and compared to a threshold (30°/sec and 80°/sec.^2^). Saccade onset was defined as two consecutive above-threshold samples for both criteria. Saccade offset was defined as the first sample of a 20-ms period of below-threshold samples.

### Data analysis

Data analysis was performed with Matlab^19–21^, using the CircStat toolbox for circular statistics^22^, and R^23^. Error was quantified as the circular distance between Gabor patch orientation and response, and relative orientation as the circular distance between the current and previous Gabor patch orientations. The raw data was first smoothed by calculating running averages across windows of 30 trials in each condition (control, identity, retinotopic, spatiotopic). A first derivative of Gaussian (DoG) was then fitted to each condition. The DoG is given by y = h + (x + b)awce − (w(x + b))^2^, where x is the relative orientation between successive trials, a the amplitude of the curve, w its width, h its height, b the intercept and c the constant √2/e^−0.5^ (the c parameter allows the parameter a to numerically match the height for ease of interpretation). In some cases, model fits to individual data could not be obtained, because the data did not conform to the DoG shape. The h and b parameters were included because they increased the probability of obtaining a model fit on the individual data, but in all cases differed little from null. (Positive h values indicate a bias towards reporting more clockwise orientations). To obtain an overall estimate in each condition in experiments 1 and 3, individual data was averaged and DoGs fitted on the aggregate data. In experiment 2, the number of trials per condition per subject differed by up to 100 trials, meaning that aggregating individual data would have averaged together windows of relative orientation that could differ widely, flattening out any serial dependence effects. Therefore, data was pooled before smoothing (see ref. ^11^ for similar methods).

Significance was assessed with permutation tests in which the x-labels (relative orientation) were randomly shuffled between trials, and a new DoG fitted on the shuffled data. This is equivalent to randomly shuffling the labels between the observed data and a null distribution of no serial dependence that has the same bias as the empirical data (parameter h). This was done 1000 times for each condition, and estimates for the a parameter obtained from the permutation distribution.
